# Serum uric acid levels and cardiorenal complication prevalence in hypertensive patients before and after the COVID-19 pandemic: a retrospective cross-sectional study from a tertiary traditional Chinese medicine hospital

**DOI:** 10.3389/fendo.2026.1830233

**Published:** 2026-05-21

**Authors:** Jin Yuan, Puxue Hua, Xuequan He, Ziyue Huo, Yanan Li, Xiaoying Yang, Rongyan Jia, Fen Li, Yin Lin

**Affiliations:** Pu’er City Hospital of Traditional Chinese Medicine, Puer, Yunnan, China

**Keywords:** cardiorenal complications, COVID-19, cross-sectional study, hypertension, hyperuricemia, interaction analysis, serum uric acid

## Abstract

**Background:**

Hyperuricemia is a recognised risk factor for cardiorenal complications in hypertensive patients. While prior studies have documented pandemic-related metabolic changes including shifts in serum uric acid (SUA) levels, no study has formally evaluated whether the post-pandemic period modifies the association between hyperuricemia and cardiorenal complication risk — that is, whether hyperuricemia and the post-pandemic period interact synergistically on cardiorenal outcomes beyond their independent effects. This interaction, assessed on both multiplicative and additive scales, remains unexamined.

**Methods:**

We conducted a retrospective cross-sectional study at Pu’er City Hospital of Traditional Chinese Medicine, comparing two non-overlapping periods: pre-pandemic (January 2017–December 2019) and post-pandemic (January 2023–March 2025). After applying predefined exclusion criteria, 3,036 hypertensive adults were included (pre-pandemic: n = 1,482; post-pandemic: n = 1,554). Hyperuricemia was defined as SUA >420 μmol/L in both men and women, in accordance with the 2019 Chinese guideline. The primary outcome was any cardiorenal complication (chronic kidney disease, heart failure, stroke, or atrial fibrillation). Fully adjusted logistic regression models were fitted, and interaction between study period and hyperuricemia was assessed on both multiplicative and additive scales, with the latter quantified using the relative excess risk due to interaction (RERI), attributable proportion (AP), and synergy index (S).

**Results:**

Post-pandemic patients had significantly higher mean SUA levels (448.70 ± 72.04 vs 422.39 ± 71.92 μmol/L; P < 0.001) and a higher prevalence of hyperuricemia (65.9% vs 52.1%; P < 0.001). The overall prevalence of any cardiorenal complication was also higher in the post-pandemic group (74.1% vs 49.1%; P < 0.001). In the fully adjusted multiplicative interaction model, hyperuricemia was independently associated with any cardiorenal complication (OR = 1.82, 95% CI: 1.39–2.39; P < 0.001), as was the post-pandemic period (OR = 1.94, 95% CI: 1.50–2.50; P < 0.001). A significant multiplicative interaction was observed between study period and hyperuricemia (interaction OR = 1.79, 95% CI: 1.27–2.53; P = 0.001). Although additive interaction measures were directionally consistent with positive synergism (RERI = 0.155; AP = 0.070; S = 1.149), the bootstrap confidence intervals included the null, indicating that evidence for additive interaction was not statistically conclusive. Findings were consistent across age and sex subgroups and across all six pre-specified sensitivity analyses.

**Conclusions:**

In this hospital-based hypertensive cohort, the post-pandemic period was associated with higher SUA levels and greater cardiorenal complication prevalence. Hyperuricemia and the post-pandemic period showed a significant multiplicative interaction on cardiorenal risk, with directionally consistent but statistically inconclusive additive synergism. These findings support routine SUA monitoring and proactive urate management in the post-pandemic care of hypertensive patients.

## 1 Introduction

Hypertension affects more than 1.28 billion adults worldwide and remains the leading modifiable risk factor for cardiovascular and renal morbidity and mortality (1–4). Beyond blood pressure elevation itself, accumulating evidence suggests that serum uric acid (SUA) is an independent and biologically plausible contributor to hypertensive target-organ damage (5, 6). As the end product of purine metabolism, uric acid has been shown to promote endothelial dysfunction, activate the renin–angiotensin–aldosterone system (RAAS), and induce NLRP3 inflammasome-mediated sterile inflammation. These mechanisms are directly implicated in the development of chronic kidney disease (CKD), heart failure (HF), stroke, and atrial fibrillation (AF) among patients with hypertension (7–9).

Large-scale epidemiological studies from China have provided robust evidence linking hyperuricemia to hypertensive target-organ damage. In the SUCCESS study, which included 9,587 participants, hyperuricemia was significantly associated with target-organ damage in patients with hypertension, even after full adjustment for conventional cardiovascular risk factors (10). Subsequent studies further demonstrated age- and sex-specific gradients in this association (11), while the H-type Hypertension Registry Study (n = 14,234) showed that hyperuricemia and elevated homocysteine exert additive effects on the risk of major adverse cardiovascular events (12). Mechanistically, uric acid may contribute to cardiovascular and renal injury through multiple pathways. Uric acid-induced oxidative stress has been shown to impair mitochondrial function in cardiomyocytes and podocytes, whereas soluble urate can directly promote vascular smooth-muscle cell proliferation and systemic endothelial dysfunction through activation of xanthine oxidase (XO) (13).

The COVID-19 pandemic profoundly disrupted metabolic health and cardiovascular risk profiles worldwide. Several pathways may link the pandemic context to elevated SUA levels. First, SARS-CoV-2 infection may increase uric acid production through enhanced cell turnover and inflammation, providing excess nucleotide substrates for xanthine oxidase (XO)-mediated urate synthesis (14). Second, COVID-19-associated acute kidney injury may reduce renal urate excretion (15). Third, pandemic-related lifestyle and psychosocial changes, including reduced physical activity, increased consumption of purine-rich diets, and stress-related neuroendocrine activation, may further contribute to SUA elevation (16). In addition, post-acute sequelae of SARS-CoV-2 infection (PASC, or “Long COVID”) are characterised by persistent low-grade inflammation, which may exacerbate pre-existing metabolic and vascular vulnerability (17–20). However, despite these biologically plausible links, it remains unclear whether the post-pandemic period modified the association between hyperuricemia and cardiorenal complication risk in patients with hypertension. To date, formal interaction analyses addressing this question are lacking. It is also important to acknowledge that pandemic-related biological mechanisms represent only one of several contributors to elevated SUA in this population. Dietary purine intake — particularly from red meat, organ meats, shellfish, and fructose-sweetened beverages — is a well-established and potentially pandemic-sensitive driver of SUA elevation, as confinement measures during 2020–2022 were associated with unfavourable dietary shifts. Furthermore, in patients with pre-existing cardiorenal disease, the relationship between hyperuricemia and organ damage is inherently bidirectional: reduced renal urate excretion in CKD and impaired haemodynamic clearance in heart failure can themselves sustain elevated SUA levels, independent of any infectious trigger. These reverse mechanisms must be considered when interpreting between-period differences in SUA within a hypertensive cohort. In this study, we use the term “cardiorenal complications” to refer collectively to four target-organ conditions — CKD, heart failure, stroke, and atrial fibrillation — that arise from the combined effects of sustained hypertension and metabolic dysregulation, and in which cardiac and renal dysfunction are closely interrelated. This framing follows the cardiorenal direction of pathophysiology, in which primary cardiac and vascular injury (driven by hypertension) leads secondarily to renal impairment, as distinct from reno-cardiac syndrome in which primary renal disease drives cardiac dysfunction. The composite outcome is operationalised as the presence of at least one of these conditions at the index visit, reflecting overall target-organ burden rather than a specific syndrome subtype.

Traditional Chinese Medicine (TCM) hospitals represent a distinctive clinical setting in China. They often care for patients with complex, multimorbid metabolic conditions who seek integrative management, including those with suboptimal response to conventional treatment. As a result, the patient population in this setting is frequently enriched for metabolic comorbidities, providing a clinically relevant high-risk cohort in which the association between uric acid and cardiorenal complications may be particularly pronounced.

Against this background, we conducted a retrospective cross-sectional study at a single Grade-A tertiary TCM hospital in China, comparing two non-overlapping periods: pre-pandemic (2017–2019) and post-pandemic (2023–2025). The primary objectives were to compare SUA levels and the prevalence of hyperuricemia between the two periods, to evaluate the independent association between hyperuricemia and any cardiorenal complication after comprehensive covariate adjustment, and to determine whether study period and hyperuricemia interacted on both multiplicative and additive scales in relation to complication risk. We further performed age-stratified and sex-stratified analyses, as well as a series of sensitivity analyses, to assess the robustness of the findings.

## 2 Materials and methods

### 2.1 Study design and setting

We conducted a retrospective cross-sectional study at Pu’er City Hospital of Traditional Chinese Medicine, a Grade-A tertiary TCM hospital located in Pu’er City, Yunnan Province, China. Data were extracted from the hospital’s Laboratory Information System (LIS), which is fully integrated with the Electronic Health Record (EHR) system and captures standardised biochemical test results from both inpatient and outpatient encounters. All serum measurements, including uric acid, lipid profiles, fasting plasma glucose, and renal function indices, were performed using a consistent automated analyser platform across both study periods, thereby supporting analytical comparability.

Two non-overlapping calendar periods were predefined: the pre-pandemic period (1 January 2017 to 31 December 2019) and the post-pandemic period (1 January 2023 to 31 March 2025). The intervening years (2020–2022) were excluded *a priori* because they represented a transitional phase marked by substantial pandemic-related healthcare and behavioural disruptions, which may have introduced temporal instability into metabolic assessments. Our intention was to compare two relatively stable periods rather than to evaluate short-term fluctuations during the transition. The choice of January 2023 as the start of the post-pandemic window was based on three considerations. First, China’s national COVID-19 control measures were formally discontinued in December 2022, after which population mobility, routine healthcare attendance, and hospital admission patterns returned to broadly pre-pandemic levels. Second, published evidence indicates that the most pronounced pandemic-related metabolic disruptions — including reductions in physical activity, dietary changes, and healthcare avoidance — were concentrated in 2020–2022, with partial recovery observed in 2023 and beyond. Third, beginning the post-pandemic window in January 2023 allowed a minimum interval of approximately one month following policy normalisation before data collection commenced. We acknowledge, however, that long-term metabolic sequelae of pandemic-related behavioural changes may persist beyond 2023 and cannot be assumed to have fully resolved. This possibility is addressed as a limitation in Section 4.6.

The study was designed and reported in accordance with the Strengthening the Reporting of Observational Studies in Epidemiology (STROBE) statement (21). Ethical approval was obtained from the Institutional Review Board of Pu’er City Hospital of Traditional Chinese Medicine (approval number: [202615]). The requirement for individual informed consent was waived because of the retrospective design and the use of de-identified data.

### 2.2 Participants

**Figure 1:**
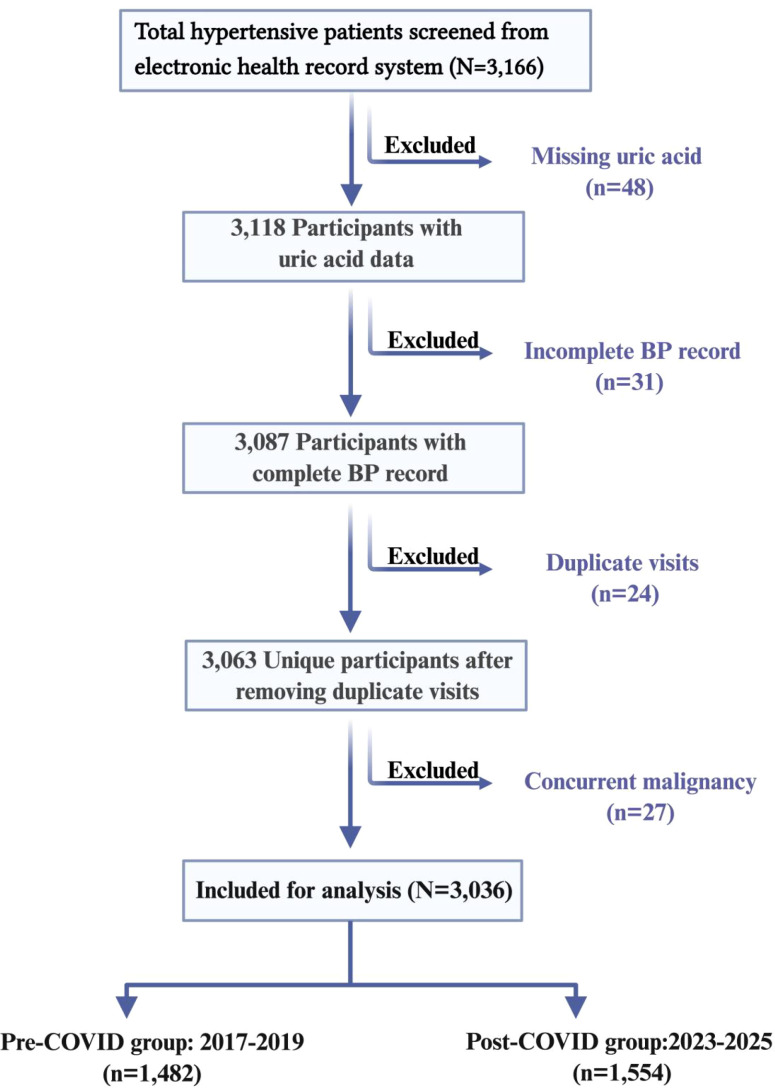
Study participant flow diagram. A total of 3,166 hypertensive patients were initially screened from the electronic health record system. After exclusion of 130 records (missing uric acid: n = 48; incomplete BP record: n = 31; concurrent malignancy: n = 27; duplicate visits: n = 24), 3,036 were included in the final analysis. Pre-pandemic group: 2017–2019 (n = 1,482); Post-pandemic group: 2023–2025 (n = 1,554).

Adults aged ≥18 years with a primary discharge or outpatient diagnosis of essential hypertension (ICD-10 code I10) were eligible for inclusion. The exclusion criteria were as follows: (1) missing fasting SUA measurement (n = 48); (2) incomplete blood pressure records (n = 31); (3) concurrent active malignancy (n = 27); and (4) duplicate visits within the same study period, for which only the index visit was retained (n = 24). The final analytic sample included 3,036 participants, comprising 1,482 in the pre-pandemic period and 1,554 in the post-pandemic period. The participant selection process is shown in **Figure 1**.

### 2.3 Exposure and covariate definitions

The primary exposure was hyperuricemia, defined as fasting SUA >420 μmol/L in both men and women, in accordance with the 2019 Chinese guidelines for hyperuricemia and gout (22, 23). SUA was measured using the uricase–peroxidase colorimetric method on a standardised automated analyser. In continuous analyses, SUA was modelled per 60 μmol/L increment, which approximated one standard deviation in the study population.

Covariates were selected *a priori* based on clinical relevance and previous literature, and included age (continuous), sex, age group (18–44, 45–64, and ≥65 years), educational attainment (four categories), marital status, body mass index (BMI; kg/m²), waist circumference (cm), smoking history (ever/never, defined as having smoked at least 100 cigarettes in a lifetime, based on self-report recorded in the structured EHR at the index visit), alcohol consumption (yes/no, defined as current regular alcohol use based on self-report), physical activity level (low/moderate/high, classified according to self-reported frequency and intensity of habitual physical activity as recorded in the clinical notes using a standardised three-category scale routinely used at this institution), hypertension duration (years), systolic blood pressure (SBP; mmHg), diastolic blood pressure (DBP; mmHg), total cholesterol (TC; mmol/L), triglycerides (TG; mmol/L), high-density lipoprotein cholesterol (HDL-C; mmol/L), low-density lipoprotein cholesterol (LDL-C; mmol/L), fasting plasma glucose (FPG; mmol/L), diabetes mellitus (yes/no), estimated glomerular filtration rate (eGFR, calculated using the CKD-EPI equation; mL/min/1.73 m²) (24), serum creatinine (μmol/L), dyslipidaemia (yes/no), history of coronary heart disease (CHD), and use of antihypertensive medications, urate-lowering therapy (ULT), aspirin, and statins.

### 2.4 Outcome definitions

The primary outcome was any cardiorenal complication, defined as a composite binary variable indicating the presence of at least one of the following conditions at or ascertainable from the index visit: (1) chronic kidney disease (CKD), defined as an estimated glomerular filtration rate (eGFR) <60 mL/min/1.73 m² persisting for ≥3 months or a urinary albumin-to-creatinine ratio (UACR) ≥30 mg/g on two consecutive measurements; (2) heart failure (HF), defined according to the Framingham Heart Study criteria and supported by echocardiographic evidence of systolic or diastolic dysfunction; (3) stroke, including ischaemic or haemorrhagic stroke confirmed by computed tomography (CT) or magnetic resonance imaging (MRI); and (4) atrial fibrillation (AF), documented on a standard 12-lead electrocardiogram or 24-hour Holter monitoring.

Secondary outcomes included each individual complication separately, the total number of complications (range, 0–4), and complication patterns categorised into 10 mutually exclusive combinations.

### 2.5 Statistical analysis

All analyses were conducted using R version 4.3.0 (R Foundation for Statistical Computing, Vienna, Austria). Continuous variables were summarised as mean ± standard deviation (SD) and compared between study periods using Welch’s two-sample t test. Categorical variables were presented as counts (percentages) and compared using Pearson’s χ² test. Because the proportion of missing data was low (<2%), complete-case analysis was performed and multiple imputation was not undertaken.

For the primary analysis, three sequential binary logistic regression models were fitted with any cardiorenal complication as the dependent variable. Model 1 was unadjusted and included hyperuricemia only. Model 2 was additionally adjusted for age, sex, educational attainment, and marital status. Model 3 was fully adjusted and additionally included all remaining covariates specified in Section 2.3. Odds ratios (ORs) and 95% confidence intervals (CIs) were estimated using the Wald method. Model performance was evaluated using the Akaike Information Criterion (AIC) and the Hosmer–Lemeshow goodness-of-fit test. Variance inflation factors (VIFs) were calculated to assess multicollinearity, with VIF <5 considered acceptable.

Interaction was assessed on both multiplicative and additive scales. For multiplicative interaction, Model 3 was extended by including a product term between study period and hyperuricemia, both coded as binary variables. Statistical significance was evaluated using a likelihood ratio test (LRT) comparing models with and without the interaction term. For additive interaction, the relative excess risk due to interaction (RERI), attributable proportion (AP), and synergy index (S) were calculated using the four-category exposure combination, with the normouricemia plus pre-pandemic group as the reference, according to the approach described by Rothman.[19] A RERI value greater than 0 was interpreted as evidence of positive additive interaction. Bootstrap 95% CIs for RERI, AP, and S were derived from 2,000 resampled datasets.

Pre-specified subgroup analyses were conducted by age group (18–44, 45–64, and ≥65 years) and by sex, with fully adjusted models fitted separately within each stratum. Between-subgroup heterogeneity was assessed using the Cochran Q statistic and the I² index; heterogeneity was considered potentially meaningful when P <0.10 or I² >50%.

To examine the robustness of the findings, six sensitivity analyses were pre-specified: (S1) exclusion of patients receiving urate-lowering therapy at baseline, to reduce treatment-related confounding; (S2) exclusion of patients with diabetes mellitus, given the close relationship between glucose metabolism and urate homeostasis; (S3) restriction to patients receiving antihypertensive medication, to improve clinical comparability; (S4) sex-stratified analyses; (S5) modelling SUA as a continuous variable per 60 μmol/L increment; and (S6) ordinal logistic regression using complication count (0–4) as the outcome under the proportional-odds assumption. All statistical tests were two-sided, and P <0.05 was considered statistically significant.

## 3 Results

### 3.1 Participant flow and baseline characteristics

Of the 3,166 hypertensive patients initially screened, 130 were excluded because of missing uric acid data (n = 48), incomplete blood pressure records (n = 31), concurrent malignancy (n = 27), or duplicate visits within the same study period (n = 24), leaving a final analytic cohort of 3,036 participants (**Figure 1**). The pre-pandemic group included 1,482 participants, and the post-pandemic group included 1,554 participants.

Baseline characteristics are summarised in **Table 1**. Compared with the pre-pandemic group, participants in the post-pandemic group were older (61.86 ± 12.51 vs 58.55 ± 12.38 years; P < 0.001) and more likely to be aged ≥65 years (41.6% vs 32.1%; P < 0.001). Sex distribution was similar between the two periods (male: 51.9% vs 53.0%; P = 0.542). Post-pandemic participants also had higher SUA levels (448.70 ± 72.04 vs 422.39 ± 71.92 μmol/L; P < 0.001), triglycerides (2.25 ± 1.12 vs 2.10 ± 1.00 mmol/L; P < 0.001), fasting plasma glucose (5.74 ± 1.20 vs 5.63 ± 1.19 mmol/L; P = 0.012), systolic blood pressure (153.07 ± 15.77 vs 151.43 ± 15.54 mmHg; P = 0.004), and longer hypertension duration (6.45 ± 4.56 vs 5.41 ± 4.22 years; P < 0.001). In contrast, estimated glomerular filtration rate was lower in the post-pandemic group (78.99 ± 12.95 vs 81.54 ± 13.05 mL/min/1.73 m²; P < 0.001). No statistically significant between-period differences were observed for body mass index, waist circumference, smoking history, alcohol consumption, physical activity level, total cholesterol, HDL-C, LDL-C, diabetes prevalence, urate-lowering therapy, aspirin use, or statin use (all P > 0.05).

**Table 1:** Baseline characteristics of study participants by study period.

Variable	Overall N = 3,036^1^	Pre-pandemic N = 1,482^1^	Post-pandemic N = 1,554^1^	p-value^2^
Age (years)	60.24 (12.55)	58.55 (12.38)	61.86 (12.51)	<0.001
Sex				0.542
Male	1,592 (52.4%)	786 (53.0%)	806 (51.9%)
Female	1,444 (47.6%)	696 (47.0%)	748 (48.1%)	
Age group				<0.001
18–44 years	345 (11.4%)	202 (13.6%)	143 (9.2%)
45–64 years	1,569 (51.7%)	805 (54.3%)	764 (49.2%)	
≥65 years	1,122 (37.0%)	475 (32.1%)	647 (41.6%)	
Education level				0.035
Primary or below	611 (20.1%)	318 (21.5%)	293 (18.9%)
Middle school	985 (32.4%)	497 (33.5%)	488 (31.4%)	
High school	857 (28.2%)	408 (27.5%)	449 (28.9%)	
College or above	583 (19.2%)	259 (17.5%)	324 (20.8%)	
Marital status				0.381
Married	2,477 (81.6%)	1,222 (82.5%)	1,255 (80.8%)
Widowed	329 (10.8%)	149 (10.1%)	180 (11.6%)	
Divorced/Single	230 (7.6%)	111 (7.5%)	119 (7.7%)	
BMI (kg/m²)	25.56 (3.42)	25.51 (3.39)	25.61 (3.45)	0.413
Waist circumference (cm)	86.20 (8.84)	86.22 (8.78)	86.19 (8.89)	0.932
Smoking history	838 (27.6%)	418 (28.2%)	420 (27.0%)	0.493
Alcohol consumption	726 (23.9%)	342 (23.1%)	384 (24.7%)	0.311
Physical activity level				0.293
Low	1,371 (45.2%)	682 (46.0%)	689 (44.3%)
Moderate	1,128 (37.2%)	554 (37.4%)	574 (36.9%)	
High	537 (17.7%)	246 (16.6%)	291 (18.7%)	
HTN duration (years)	5.94 (4.43)	5.41 (4.22)	6.45 (4.56)	<0.001
SBP (mmHg)	152.27 (15.68)	151.43 (15.54)	153.07 (15.77)	0.004
DBP (mmHg)	93.74 (11.77)	93.21 (11.87)	94.25 (11.64)	0.015
Serum uric acid (μmol/L)	435.86 (73.16)	422.39 (71.92)	448.70 (72.04)	<0.001
Hyperuricemia				<0.001
Normal	1,240 (40.8%)	710 (47.9%)	530 (34.1%)
Hyperuricemia	1,796 (59.2%)	772 (52.1%)	1,024 (65.9%)	
Total cholesterol (mmol/L)	5.00 (0.94)	5.01 (0.94)	5.00 (0.93)	0.735
Triglycerides (mmol/L)	2.18 (1.06)	2.10 (1.00)	2.25 (1.12)	<0.001
HDL-C (mmol/L)	1.21 (0.25)	1.22 (0.25)	1.21 (0.25)	0.153
LDL-C (mmol/L)	2.83 (1.06)	2.86 (1.07)	2.80 (1.06)	0.130
Fasting glucose (mmol/L)	5.69 (1.20)	5.63 (1.19)	5.74 (1.20)	0.012
Diabetes mellitus	666 (21.9%)	304 (20.5%)	362 (23.3%)	0.071
eGFR (mL/min/1.73m²)	80.24 (13.06)	81.54 (13.05)	78.99 (12.95)	<0.001
Serum creatinine (μmol/L)	87.40 (18.44)	85.52 (17.96)	89.19 (18.72)	<0.001
Dyslipidaemia	2,564 (84.5%)	1,222 (82.5%)	1,342 (86.4%)	0.004
History of CHD	116 (3.8%)	51 (3.4%)	65 (4.2%)	0.332
Antihypertensive medication	2,249 (74.1%)	1,111 (75.0%)	1,138 (73.2%)	0.294
Urate-lowering therapy	651 (21.4%)	310 (20.9%)	341 (21.9%)	0.520
Aspirin use	971 (32.0%)	475 (32.1%)	496 (31.9%)	0.968
Statin use	1,461 (48.1%)	691 (46.6%)	770 (49.5%)	0.115

^1^ Mean (SD); n (%).

^2^ Welch Two Sample t-test; Pearson's Chi-squared test.

Data are mean ± SD for continuous variables (compared by two-sample Welch t-test) and n (%) for categorical variables (compared by Pearson χ² test). BMI, body mass index; HTN, hypertension; SBP, systolic blood pressure; DBP, diastolic blood pressure; SUA, serum uric acid; TC, total cholesterol; TG, triglycerides; HDL-C, high-density lipoprotein cholesterol; LDL-C, low-density lipoprotein cholesterol; FPG, fasting plasma glucose; eGFR: estimated glomerular filtration rate (CKD-EPI equation); CHD, coronary heart disease; ULT, urate-lowering therapy.

### 3.2 Serum uric acid levels and hyperuricemia prevalence

**Figure 2:**
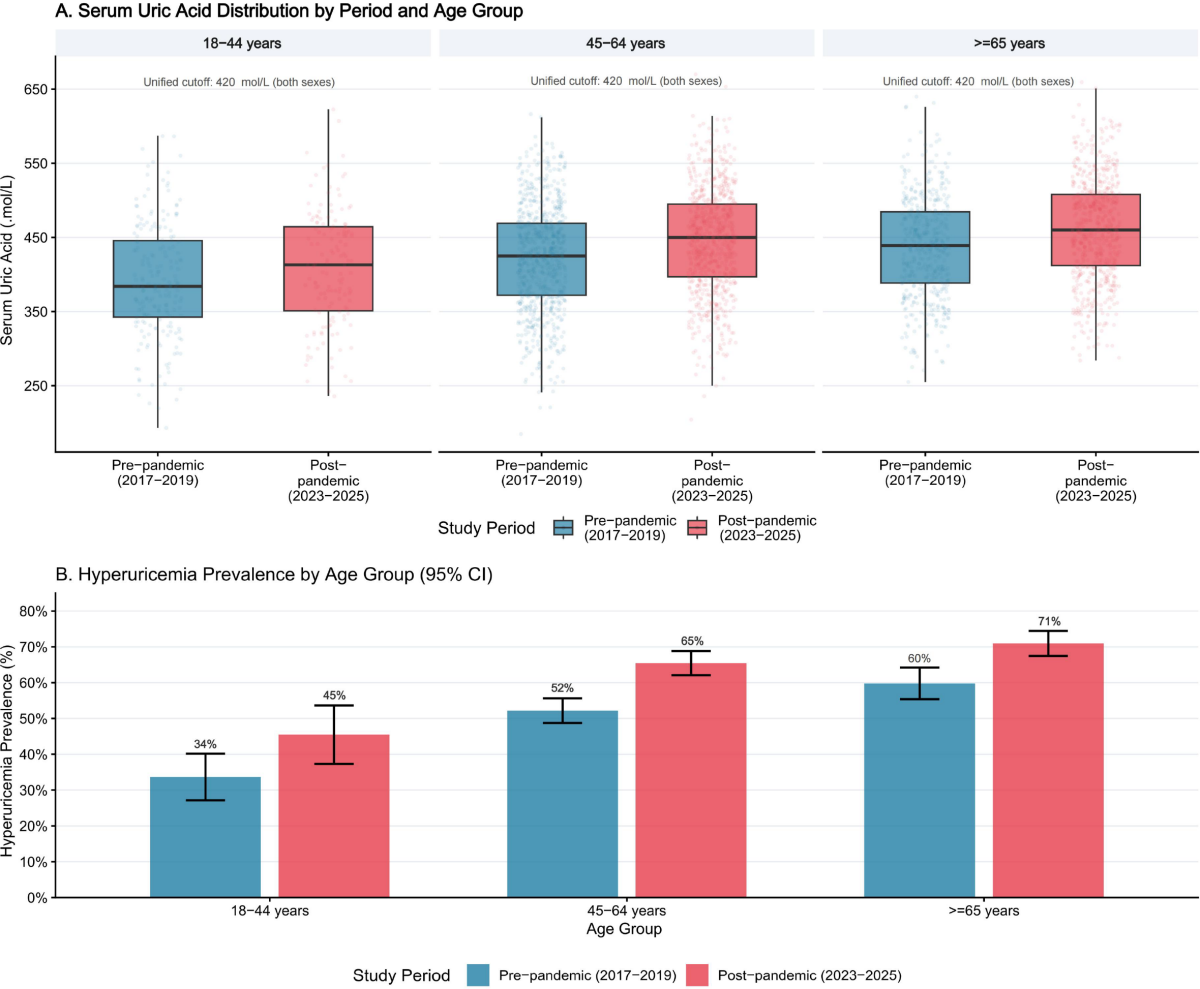
Serum uric acid distribution and hyperuricemia prevalence across study periods and age groups. **(A)** Box-and-jitter plots of SUA (μmol/L) stratified by period and age group. The dashed line indicates the unified hyperuricemia threshold (>420 μmol/L for both sexes), in accordance with the 2019 Chinese guideline. **(B)** Hyperuricemia prevalence (%) by age group in each period, with 95% confidence interval error bars. Blue: Pre-pandemic; Red: Post-pandemic.

SUA levels and hyperuricemia prevalence across study periods and age groups are presented in **Table 2** and **Figure 2**. Mean SUA was 26.3 μmol/L higher in the post-pandemic period than in the pre-pandemic period (95% CI: 21.7–30.9; P < 0.001). The prevalence of hyperuricemia increased from 52.1% in the pre-pandemic period to 65.9% in the post-pandemic period, corresponding to an absolute risk difference of 13.8 percentage points (χ² = 64.1; P < 0.001).

**Table 2:** Uric acid distribution by period and age group.

Characteristic	Pre-pandemic				Post-pandemic			
	18–44 years N = 202^1^	45–64 years N = 805^1^	≥65 years N = 475^1^	p-value^2^	18–44 years N = 143^1^	45–64 years N = 764^1^	≥65 years N = 647^1^	p-value^2^
Serum uric acid (μmol/L)	391 (76)	422 (71)	437 (68)	<0.001	409 (78)	446 (71)	461 (69)	<0.001
Hyperuricemia				<0.001				<0.001
Normal	134 (66%)	385 (48%)	191 (40%)		78 (55%)	264 (35%)	188 (29%)	
Hyperuricemia	68 (34%)	420 (52%)	284 (60%)		65 (45%)	500 (65%)	459 (71%)	

^1^ Mean (SD); n (%).

^2^ Kruskal-Wallis rank sum test; Pearson's Chi-squared test.

Data are mean (SD) for continuous variables and n (%) for categorical variables. P-values for continuous variables computed by Kruskal–Wallis test; for categorical variables by Pearson χ² test.

Age-stratified analyses showed that the increase in hyperuricemia prevalence was observed across all age groups: from 34% to 45% among participants aged 18–44 years (absolute difference, 11 percentage points), from 52% to 65% among those aged 45–64 years (absolute difference, 13 percentage points), and from 60% to 71% among those aged ≥65 years (absolute difference, 11 percentage points). Mean SUA was highest in the oldest age group in both periods (≥65 years: 437 ± 68 μmol/L in the pre-pandemic period vs 461 ± 69 μmol/L in the post-pandemic period; **Figures 2A, B**).

### 3.3 Cardiorenal complication prevalence

Complication rates stratified by study period and uric acid category are presented in **Table 3**. Overall, the prevalence of any cardiorenal complication was higher in the post-pandemic group than in the pre-pandemic group (74.1% vs 49.1%; P < 0.001). Similar between-period increases were observed for individual complications overall, including CKD (51.7% vs 27.3%), heart failure (39.4% vs 21.0%), stroke (27.7% vs 13.4%), and atrial fibrillation (15.8% vs 7.4%) (all P < 0.001).

**Table 3:** Complication prevalence by period and uric acid category.

Characteristic	Normal			Hyperuricemia		
	Pre-pandemic N = 710^1^	Post-pandemic N = 530^1^	p-value^2^	Pre-pandemic N = 772^1^	Post-pandemic N = 1,024^1^	p-value^2^
Chronic kidney disease	143 (20%)	181 (34%)	<0.001	262 (34%)	623 (61%)	<0.001
Heart failure	116 (16%)	140 (26%)	<0.001	195 (25%)	473 (46%)	<0.001
Stroke	69 (9.7%)	105 (20%)	<0.001	130 (17%)	325 (32%)	<0.001
Atrial fibrillation	36 (5.1%)	64 (12%)	<0.001	74 (9.6%)	181 (18%)	<0.001
Any complication	270 (38%)	298 (56%)	<0.001	458 (59%)	854 (83%)	<0.001
Number of complications			<0.001			<0.001
0	440 (62%)	232 (44%)		314 (41%)	170 (17%)	
1	189 (27%)	157 (30%)		300 (39%)	339 (33%)	
2	70 (9.9%)	95 (18%)		118 (15%)	318 (31%)	
3	9 (1.3%)	41 (7.7%)		35 (4.5%)	161 (16%)	
4	2 (0.3%)	5 (0.9%)		5 (0.6%)	36 (3.5%)	

^1^ n (%).

^2^ Pearson's Chi-squared test

Data expressed as n (%). P-values by Pearson χ² test comparing Pre-pandemic versus Post-pandemic within each uric acid category. CKD, chronic kidney disease; HF, heart failure; AF, atrial fibrillation.

**Figure 3:**
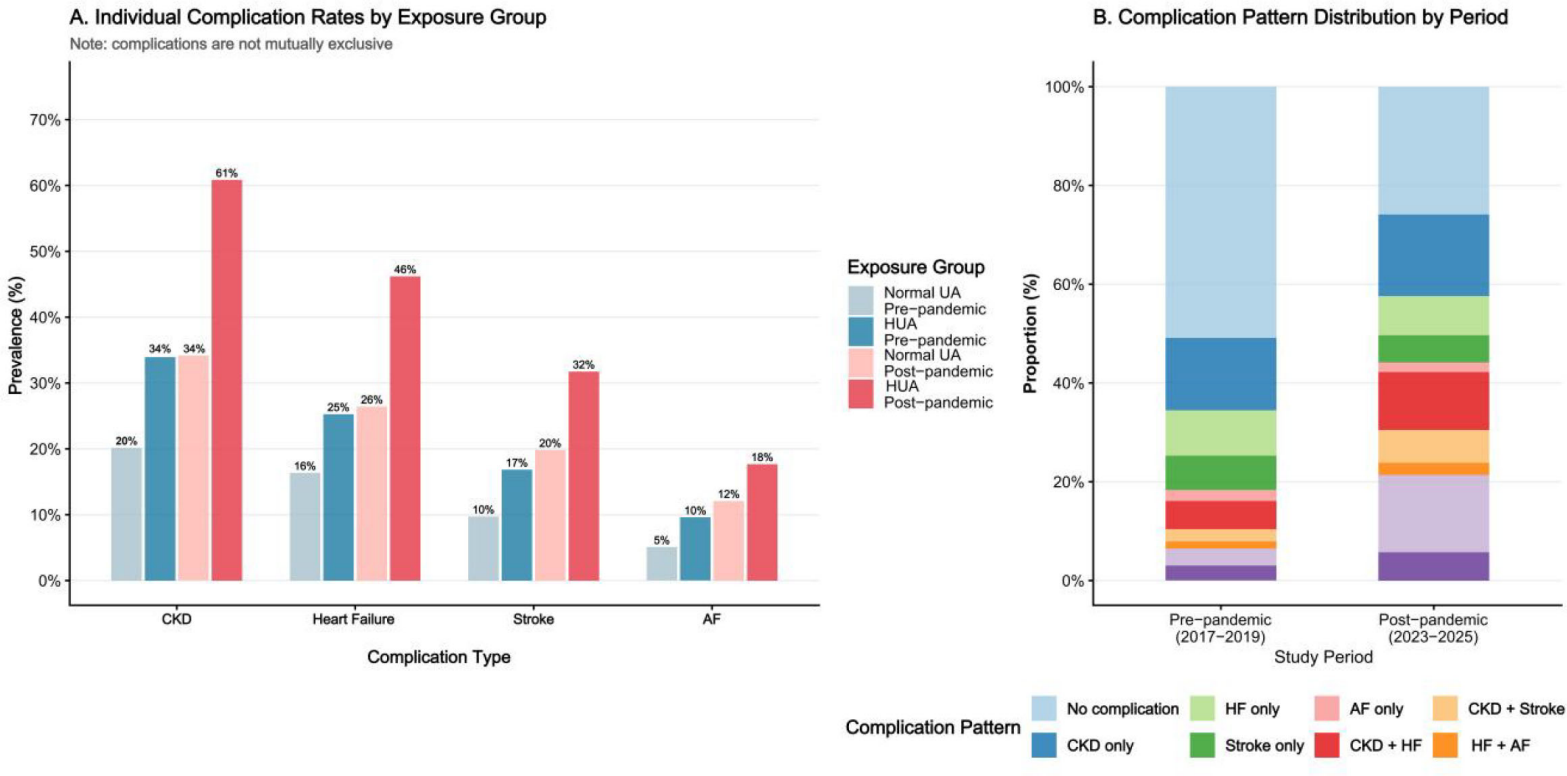
Cardiorenal complication patterns by period and uric acid category. **(A)** Stacked bar chart of individual complication prevalence for each of the four exposure groups. **(B)** Complication pattern distribution (proportional stacked bar chart) by study period. CKD, chronic kidney disease; HF, heart failure; AF, atrial fibrillation; HUA, hyperuricemia.

Among participants with hyperuricemia, the prevalence of any cardiorenal complication increased from 59.3% in the pre-pandemic period to 83.4% in the post-pandemic period, corresponding to an absolute difference of 24.1 percentage points (P < 0.001). Among those without hyperuricemia, the prevalence also increased, although to a lesser extent, from 38.0% to 56.2% (absolute difference, 18.2 percentage points; P < 0.001). Compared with the reference group of normouricemic participants in the pre-pandemic period, the absolute excess prevalence in participants with both hyperuricemia and the post-pandemic exposure profile was 45.4 percentage points. In addition, complication multiplicity, defined as the presence of two or more concurrent conditions, was observed in 50.5% of post-pandemic participants with hyperuricemia, compared with 20.1% of pre-pandemic participants with hyperuricemia (**Figure 3**).

### 3.4 Association between hyperuricemia and cardiorenal complications: logistic regression

The fully adjusted associations between serum uric acid and cardiorenal complications stratified by study period are presented in **Table 4**. In the pre-pandemic period, hyperuricemia was independently associated with higher odds of any cardiorenal complication (OR = 1.70, 95% CI: 1.27–2.29; P < 0.001), CKD (OR = 1.61, 95% CI: 1.16–2.24; P = 0.005), and heart failure (OR = 1.45, 95% CI: 1.02–2.06; P = 0.040), while the associations with stroke (OR = 1.20, 95% CI: 0.79–1.83; P = 0.395) and AF (OR = 1.63, 95% CI: 0.94–2.85; P = 0.081) did not reach statistical significance. In the post-pandemic period, the corresponding associations were generally stronger, with hyperuricemia associated with any complication (OR = 3.55, 95% CI: 2.50–5.06; P < 0.001), CKD (OR = 2.51, 95% CI: 1.86–3.39; P < 0.001), and heart failure (OR = 1.80, 95% CI: 1.34–2.41; P < 0.001), whereas the associations with stroke (OR = 1.38, 95% CI: 1.00–1.91; P = 0.051) and AF (OR = 1.34, 95% CI: 0.92–1.98; P = 0.134) again did not reach conventional statistical significance.

**Table 4:** Association of serum uric acid with cardiorenal complications stratified by study period: fully adjusted logistic regression (model 3).

		Any complication		CKD		Heart failure		Stroke		AF	
Group	Characteristic	OR(95% CI)	*P*-value	OR(95% CI)	*P*-value	OR(95% CI)	*P*-value	OR(95% CI)	*P*-value	OR(95% CI)	*P*-value
Pre-pandemic (n = 1482)											
Hyperuricemia (Category)											
	Normal	ref									
	Hyperuricemia	2.6(1.94, 3.52)	<0.001	1.61(1.16, 2.24)	<0.001	1.45(1.02, 2.06)	<0.001	1.20(0.79, 1.83)	0.027	1.63(0.94, 2.85)	0.002
SUA per 60 μmol/L	SUA per 60 μmol/L	1.4(1.21, 1.62)	<0.001	1.45(1.23, 1.70)	<0.001	1.31(1.10, 1.55)	0.002	1.16(0.95, 1.42)	0.146	1.13(0.87, 1.45)	0.357
Post-pandemic (n = 1554)											
	Normal	ref									
	Hyperuricemia	3.55(2.50, 5.06)	<0.001	2.51(1.86, 3.39)	<0.001	3.4(2.33, 5.03)	<0.001	1.38(1.00, 1.91)	<0.001	1.34(0.92, 1.98)	0.007
SUA per 60 μmol/L	SUA per 60 μmol/L	1.76(1.49, 2.09)	<0.001	1.54(1.34, 1.77)	<0.001	1.26(1.11, 1.45)	<0.001	1.14(0.98, 1.32)	0.089	1.21(1.01, 1.43)	0.034

1All estimates from Model 3 (fully adjusted). OR, odds ratio; CI, 95% confidence interval. Covariates: age, sex, education, marital status, BMI, waist circumference, smoking, alcohol, physical activity, HTN duration, SBP, TG, HDL-C, LDL-C, fasting glucose, diabetes, eGFR, dyslipidaemia, CHD history, antihypertensive medication, urate-lowering therapy, aspirin, statin, DBP, TC, serum creatinine. Full three-model estimates in **Supplementary Table S1**.

CI, Confidence Interval, OR, Odds Ratio.

When SUA was modelled as a continuous variable per 60 μmol/L increment, higher SUA was also associated with greater odds of several complications in both periods. In the pre-pandemic period, each 60 μmol/L increase in SUA was associated with any complication (OR = 1.40, 95% CI: 1.21–1.62; P < 0.001), CKD (OR = 1.45, 95% CI: 1.23–1.70; P < 0.001), and heart failure (OR = 1.31, 95% CI: 1.10–1.55; P = 0.002), but not stroke or AF. In the post-pandemic period, each 60 μmol/L increase in SUA was associated with any complication (OR = 1.76, 95% CI: 1.49–2.09; P < 0.001), CKD (OR = 1.54, 95% CI: 1.34–1.77; P < 0.001), heart failure (OR = 1.26, 95% CI: 1.11–1.45; P < 0.001), and AF (OR = 1.21, 95% CI: 1.01–1.43; P = 0.034), whereas the association with stroke did not reach statistical significance.

### 3.5 Interaction between study period and hyperuricemia

**Table 5** presents the interaction analysis results. In the fully adjusted multiplicative interaction model, hyperuricemia was associated with higher odds of any cardiorenal complication (OR = 1.82, 95% CI: 1.39–2.39; P < 0.001), and the post-pandemic period was also independently associated with higher odds of complications (OR = 1.94, 95% CI: 1.50–2.50; P < 0.001). Importantly, the hyperuricemia × post-pandemic interaction term was statistically significant (OR = 1.79, 95% CI: 1.27–2.53; P = 0.001), and this was confirmed by the likelihood ratio test (LRT χ² = 10.87, P < 0.001), indicating that the association between hyperuricemia and cardiorenal complications was stronger in the post-pandemic period than in the pre-pandemic period.

**Table 5:** Interaction analysis between study period and hyperuricemia on the risk of any cardiorenal complication.

Analysis layer	Parameter	Estimate	95% CI	*P* value
Observed complication prevalence by exposure group				
	Normouricemia + Pre-pandemic (reference)	270/710 (38.0%)	—	—
	Hyperuricemia + Pre-pandemic	458/772 (59.3%)	—	—
	Normouricemia + Post-pandemic	298/530 (56.2%)	—	—
	Hyperuricemia + Post-pandemic	854/1024 (83.4%)	—	—
Multiplicative interaction (fully adjusted model)				
	Hyperuricemia (vs normouricemia)	1.82	1.39–2.39	<0.001
	Post-pandemic period (vs pre-pandemic period)	1.94	1.50–2.50	<0.001
	Hyperuricemia × Post-pandemic	1.79	1.27–2.53	<0.001
Additive interaction measures				
	RERI	0.155	-0.023–0.324	—
	AP	0.070	-0.010–0.152	—
	S	1.149	0.983–1.574	—

a. Covariates: age, sex, education, marital status, BMI, waist circumference, smoking, alcohol, physical activity, HTN duration, SBP, TG, HDL-C, LDL-C, fasting glucose, diabetes, eGFR, dyslipidaemia, CHD history, antihypertensive medication, urate-lowering therapy, aspirin, statin,DBP, TC,.serum creatinine. Total observations: n = 3,036; AIC = 3,134.

b. Likelihood ratio test for the interaction term: χ² = 10.87, df = 1, P < 0.001.

c. Additive interaction measures were estimated with bootstrap 95% confidence intervals based on 2,000 resampled datasets. RERI > 0 and S > 1 indicate positive additive interaction.

AP, attributable proportion due to interaction; AIC, Akaike information criterion; CI, confidence interval; HDL-C, high-density lipoprotein cholesterol; RERI, relative excess risk due to interaction; S, synergy index.

Additive interaction analysis based on the four exposure groups showed complication prevalences of 38.0% in the normouricemia plus pre-pandemic group, 59.3% in the hyperuricemia plus pre-pandemic group, 56.2% in the normouricemia plus post-pandemic group, and 83.4% in the hyperuricemia plus post-pandemic group. The corresponding additive interaction measures were RERI = 0.155 (95% CI: −0.023–0.324), AP = 0.070 (95% CI: −0.010–0.152), and S = 1.149 (95% CI: 0.983–1.409). Although these estimates were directionally consistent with positive additive interaction, the bootstrap confidence intervals included the null, indicating that the evidence for additive synergism was not statistically conclusive.

### 3.6 Subgroup analyses

**Figure 4:**
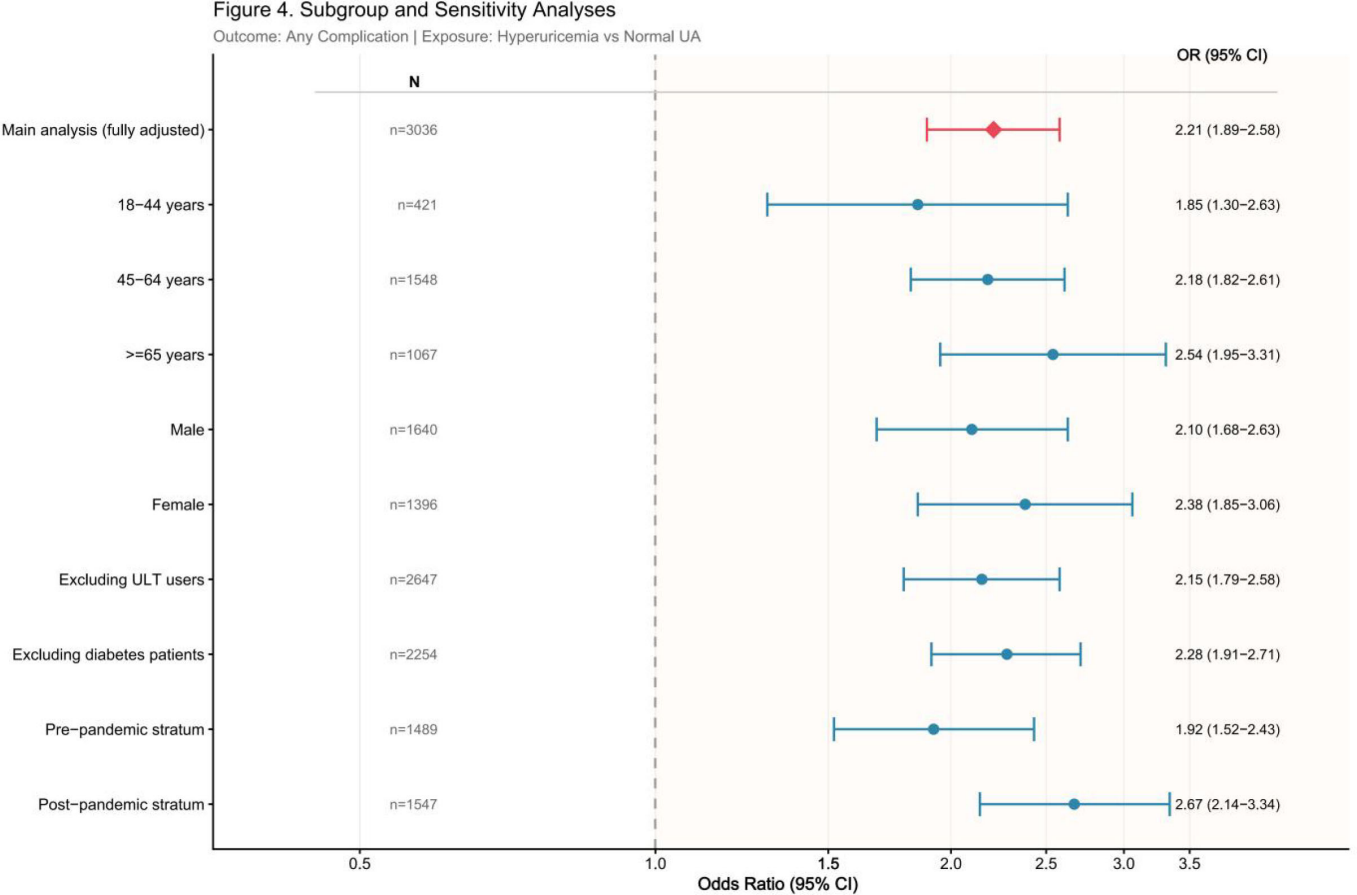
Forest plot of subgroup and sensitivity analyses. The outcome is any cardiorenal complication; the exposure is hyperuricemia versus normal SUA. All subgroup models employed the same fully adjusted covariate set as Model 3. The red diamond represents the main analysis estimate; blue circles represent subgroup-specific estimates. OR, odds ratio; CI, confidence interval.

Subgroup analyses stratified by age group, sex, and study period are presented in **Figure 4**. In age-stratified analyses, hyperuricemia was significantly associated with any cardiorenal complication across all age groups in the fully adjusted models. The fully adjusted ORs were 4.17 (95% CI: 1.87–9.30; P < 0.001) for participants aged 18–44 years, 2.01 (95% CI: 1.51–2.67; P < 0.001) for those aged 45–64 years, and 2.54 (95% CI: 1.66–3.90; P < 0.001) for those aged ≥65 years.

Sex-stratified analyses showed a similar pattern. Hyperuricemia was associated with higher odds of any cardiorenal complication in both men (OR = 4.07, 95% CI: 2.90–5.70; P < 0.001) and women (OR = 1.55, 95% CI: 1.14–2.10; P < 0.001).

### 3.7 Sensitivity analyses

Across all six pre-specified sensitivity analyses, the association between hyperuricemia and any cardiorenal complication remained directionally consistent and statistically significant (**Supplementary Table S1**). Excluding patients receiving urate-lowering therapy and those with diabetes mellitus yielded effect estimates similar to the primary analysis, with ORs of 2.629 (95% CI: 2.058–3.358) and 2.146 (95% CI: 1.675–2.749), respectively. Restricting the analysis to antihypertensive medication users also produced a nearly identical estimate (OR = 2.484, 95% CI: 1.911–3.231). Furthermore, sex-stratified sensitivity analyses confirmed that the associations remained robust in both men and women (all P < 0.001, **Supplementary Table S1**). In addition, ordinal logistic regression using complication count as the outcome confirmed the robustness of the findings (proportional-odds OR = 2.009, 95% CI: 1.675–2.413).

## 4 Discussion

In this retrospective cross-sectional study of 3,036 hypertensive patients treated at a tertiary Traditional Chinese Medicine hospital, we identified three principal findings. First, compared with the pre-pandemic period, the post-pandemic period was associated with higher mean SUA levels (+26.3 μmol/L) and a higher prevalence of hyperuricemia (+13.8 percentage points). Second, in the fully adjusted interaction model, hyperuricemia was independently associated with higher odds of any cardiorenal complication (OR = 1.82), while the post-pandemic period was also independently associated with increased odds (OR = 1.94). Third, hyperuricemia and study period showed significant multiplicative interaction (interaction OR = 1.79, 95% CI: 1.27–2.53; P = 0.001), although additive interaction measures (RERI = 0.155, AP = 0.070, S = 1.149) were directionally consistent with positive synergism but did not reach statistical significance.

### 4.1 Post-pandemic increase in serum uric acid: potential mechanisms and clinical implications

The higher SUA levels observed in the post-pandemic period are broadly consistent with emerging evidence suggesting adverse metabolic changes following the pandemic (25, 26). The underlying mechanisms are likely multifactorial. At the biological level, SARS-CoV-2 infection may promote urate accumulation through inflammation-related cell injury and increased nucleotide turnover, thereby increasing substrate availability for xanthine oxidase-mediated urate production (27). In addition, COVID-19-associated renal injury may impair urate excretion, at least in some individuals, and this effect may persist beyond the acute phase in those with incomplete renal recovery (28).

Beyond direct infection-related effects, the broader post-pandemic environment may also have contributed to higher uric acid levels through behavioural and metabolic pathways (29). Reduced physical activity, dietary shifts, and psychosocial stress have all been linked to worsening cardiometabolic health, and may plausibly promote hyperuricemia. In our cohort, the post-pandemic group also showed higher triglyceride and fasting plasma glucose levels, supporting the presence of an adverse metabolic profile during this period. Notably, the post-pandemic period remained independently associated with any cardiorenal complication in the fully adjusted interaction model (OR = 1.94, 95% CI: 1.50–2.50), suggesting that the observed increase in complication burden was not fully explained by measured metabolic covariates alone (30).

Age-stratified analyses provide additional context. Although the rise in hyperuricemia prevalence was evident across all age groups, older participants had the highest absolute SUA levels in both periods. Moreover, among participants aged ≥65 years, hyperuricemia remained strongly associated with any cardiorenal complication (OR = 2.54, 95% CI: 1.66–3.90) (31). This pattern may reflect the greater vulnerability of older hypertensive patients, who often have reduced renal reserve and a higher burden of underlying cardiovascular and renal comorbidity, making them more susceptible to additional urate-related injury.

### 4.2 Independent association between hyperuricemia and cardiorenal complications

In the fully adjusted interaction model, hyperuricemia was independently associated with higher odds of any cardiorenal complication (OR = 2.35). This estimate is broadly consistent with, though at the higher end of, those reported in several large Chinese epidemiological studies (12), and also exceeds the pooled estimate reported in a recent meta-analysis of CKD risk (31, 32). Several factors may contribute to this difference. First, our study was conducted in a tertiary TCM hospital, where referral patterns may enrich for patients with more complex metabolic and cardiovascular comorbidity. This interpretation is supported by the high prevalence of hyperuricemia in our cohort (59.2%), which exceeds that reported in most community-based hypertensive populations. Second, our primary outcome was a composite endpoint encompassing four cardiorenal complications, which may capture a broader burden of disease than analyses focused on a single outcome. Third, the application of the unified 420 μmol/L threshold for both sexes — as recommended by the 2019 Chinese guideline — means that women with SUA in the 361–420 μmol/L range are classified as normouricaemic in this study, whereas prior studies using sex-specific thresholds would have classified them as hyperuricaemic. This reclassification concentrates genuinely high-SUA individuals in the hyperuricaemia group, potentially strengthening the observed association. Fourth, although study period was included in the model, residual confounding related to unmeasured post-pandemic exposures cannot be excluded. Finally, SUA was assessed at a single time point, whereas longitudinal studies using repeated measurements may yield more conservative estimates because of reduced regression dilution.

The pattern of association across individual complications also appears biologically plausible. In our fully adjusted period-stratified analyses, the strongest and statistically significant associations were observed for CKD and heart failure, whereas the associations with stroke and AF did not reach conventional statistical significance in either period. This pattern is consistent with current understanding of uric acid-related cardiorenal injury. Experimental and clinical evidence suggests that uric acid and xanthine oxidase activity may contribute to renal microvascular dysfunction, impaired autoregulation, and reduced glomerular filtration through oxidative stress, nitric oxide depletion, and activation of the renin–angiotensin–aldosterone system (33). In the heart, hyperuricemia has been linked to inflammation, myocardial fibrosis, and diastolic dysfunction, which may help explain its association with heart failure (34). Uric acid has also been implicated in endothelial dysfunction, atherosclerotic progression, and structural remodelling, providing plausible biological links to stroke and atrial fibrillation, although the statistical evidence for these associations was not significant in the present study, possibly reflecting insufficient statistical power for these less prevalent endpoints. The sex-stratified analyses revealed a notably larger association in men (OR = 4.07) than in women (OR = 1.55). This difference warrants interpretive caution. Under the unified diagnostic threshold of 420 μmol/L, women with SUA in the 361–420 μmol/L range — who would previously have been classified as hyperuricaemic under sex-specific criteria — are now classified as normouricaemic. This reclassification concentrates the most severely hyperuricaemic women in the HUA group and the lowest-SUA women in the Normal group, potentially amplifying the apparent OR for women. Conversely, the male threshold is unchanged, so the male subgroup estimate is less affected by this reclassification. This methodological effect, rather than true biological sex-modification alone, may partly explain the magnitude of the between-sex difference observed.

### 4.3 Synergistic interaction: biological plausibility and clinical significance

A key finding of this study was the significant multiplicative interaction between hyperuricemia and study period. The multiplicative interaction term was statistically significant (OR = 1.79, 95% CI: 1.27–2.53; P = 0.001), indicating that the relative association between hyperuricemia and cardiorenal complications was stronger in the post-pandemic period than in the pre-pandemic period. Additive interaction measures were directionally consistent with positive synergism (RERI = 0.155, AP = 0.070, and S = 1.149); however, the bootstrap confidence intervals crossed the null, and these estimates should therefore be interpreted with caution. From a clinical and public health perspective, the confirmed multiplicative interaction suggests that hyperuricaemic patients in the post-pandemic period face a disproportionately elevated relative risk, even if the evidence for absolute additive synergism remains inconclusive.

The biological basis for this interaction is plausible. Post-pandemic states have been associated with persistent low-grade inflammation, endothelial dysfunction, and metabolic disturbance in at least a subset of patients (25). Hyperuricemia, in turn, is linked to oxidative stress, xanthine oxidase activation, endothelial injury, and NLRP3 inflammasome signalling. It is therefore biologically plausible that these processes may reinforce one another when they co-occur, thereby amplifying cardiorenal vulnerability beyond the effect of either factor alone. In addition, pandemic-related behavioural and metabolic changes may have further contributed to this interaction by worsening underlying susceptibility in hypertensive patients. Taken together, these considerations support heightened clinical attention to hyperuricemic patients in the post-pandemic period, particularly those with pre-existing cardiovascular or renal risk.

### 4.4 The TCM hospital context: strengths and interpretive caution

The TCM hospital setting is an important contextual feature of this study and should be considered in interpreting the findings. On the one hand, this setting provided access to a clinically complex hypertensive population with detailed biochemical measurements and a relatively rich set of covariates, allowing more comprehensive adjustment than is often feasible in routine primary care datasets. In addition, the high burden of metabolic comorbidity in this referral-based population may have increased the ability to detect clinically meaningful associations between hyperuricemia and cardiorenal complications.

On the other hand, the findings should be interpreted within this high-risk institutional context. The high prevalence of hyperuricemia in our cohort (59.2%) and the relatively large adjusted effect estimates may not be directly generalisable to community-based hypertensive populations with lower baseline metabolic risk. In addition, as this was a TCM hospital, it is possible that some patients received integrative treatments alongside conventional therapy; however, detailed records of such treatments were not systematically available in the EHR in a form suitable for analysis, and their potential effects on uric acid metabolism represent an unmeasured source of uncertainty. Future studies with more complete treatment records, including documentation of integrative therapies, are needed to determine the extent to which these findings apply beyond tertiary TCM practice.

### 4.5 Comparison with existing literature

Our findings add to the existing literature in several ways. First, previous studies in Chinese hypertensive populations established the cross-sectional association between hyperuricemia and hypertensive target-organ damage under pre-pandemic conditions. In contrast, the present study examined whether study period modified this association and evaluated interaction on both multiplicative and additive scales within a single well-characterised cohort. Second, although recent work summarised potential biological links between COVID-19 and elevated uric acid levels, empirical data connecting these mechanisms to downstream cardiorenal complications at the patient level remain limited. Our study helps address this gap by providing clinical outcome data from 3,036 hypertensive patients with extensive covariate adjustment. Third, whereas prior interaction analyses in hypertension research have largely focused on multiplicative interaction, we additionally assessed additive interaction using RERI and AP, thereby providing a complementary epidemiologic perspective on whether the joint association of hyperuricemia and the post-pandemic period exceeded the sum of their individual associations.

### 4.6 Limitations

Several limitations should be acknowledged. First, the retrospective cross-sectional design precludes causal inference. The temporal sequence between hyperuricemia and cardiorenal complications cannot be established, and reverse causation remains possible, particularly for CKD, in which reduced renal urate excretion may independently sustain elevated SUA levels. Second, although a broad range of covariates was included, residual confounding by unmeasured factors cannot be excluded. Most importantly, individual-level COVID-19 infection history, illness severity, and post-acute sequelae were not available in the hospital records; the observed between-period differences in SUA and complication prevalence therefore reflect a temporal contrast rather than a confirmed post-infection effect, and causal attribution to COVID-19 is not warranted. Third, dietary purine intake was not measured, as structured dietary assessments were not routinely recorded in the EHR. This is a recognised limitation of registry-based retrospective studies, and future prospective work should incorporate validated dietary assessment instruments. Fourth, lifestyle variables — including smoking history, alcohol consumption, and physical activity — were derived from self-report recorded in the structured EHR and are therefore relatively coarse measures that may not fully capture behavioural changes occurring across the two study periods. Fifth, SUA was measured at a single time point, precluding assessment of long-term urate exposure and introducing the possibility of regression dilution. Sixth, antihypertensive medication use was captured as a binary covariate without information on drug class, dosage, or duration; the potential uricogenic effect of diuretics and other agents therefore cannot be fully accounted for. Seventh, complication ascertainment relied on structured electronic health record data and may have been affected by incomplete coding or under-recording. Eighth, the unequal sample sizes across periods (pre-pandemic: n = 1,482; post-pandemic: n = 1,554), combined with baseline differences in age and hypertension duration, preclude simple unadjusted comparison and reinforce the necessity of the fully adjusted analytical approach. Finally, the single-centre design and the distinctive case-mix of a tertiary TCM hospital may limit generalisability to broader hypertensive populations, particularly those in primary care or community-based settings. Future prospective studies incorporating repeated SUA measurements, individual-level COVID-19 exposure data, structured dietary assessment, and standardised adjudication of cardiorenal outcomes are needed to clarify temporality and refine risk stratification.

## 5 Conclusions

In this retrospective cross-sectional study of hypertensive patients treated at a tertiary TCM hospital, the post-pandemic period was associated with higher SUA levels and a greater prevalence of hyperuricemia than the pre-pandemic period. Hyperuricemia was independently associated with higher odds of any cardiorenal complication, and this association was stronger in the post-pandemic period, as reflected by significant multiplicative interaction (interaction OR = 1.79; P = 0.001). Additive interaction measures were directionally consistent with synergism but did not reach statistical significance (RERI = 0.155, 95% CI: −0.023–0.324). These findings suggest that closer SUA monitoring may be warranted in hypertensive patients during the post-pandemic period, and that hyperuricemia may represent an important marker of increased cardiorenal vulnerability. Further prospective studies are needed to determine whether targeted urate-lowering strategies can reduce complication burden in this population.

## Data Availability

The raw data supporting the conclusions of this article will be made available by the authors, without undue reservation.
