# Supplementary material for: Serum uric acid levels and cardiorenal complication prevalence in hypertensive patients before and after the COVID-19 pandemic: a retrospective cross-sectional study from a tertiary traditional Chinese medicine hospital
Source: Front Endocrinol (Lausanne). 2026 May 21;17:1830233. doi: 10.3389/fendo.2026.1830233 (PMC13233390; doi:10.3389/fendo.2026.1830233)
Supplement: Supplementary file 1 [file Table1.docx]

**Supplementary Table S1.** Full covariate estimates from the fully adjusted logistic regression model (Model 3, combined periods)

| **Characteristic** | **OR** | **95% CI** | **p-value** |
| --- | --- | --- | --- |
| Hyperuricemia (vs Normal UA) |  |  |  |
| Normal | — | — |  |
| Hyperuricemia | 3.33 | 2.66, 4.18 | **<0.001** |
| Post-COVID (vs Pre-COVID) |  |  |  |
| Pre-COVID | — | — |  |
| Post-COVID | 2.67 | 2.24, 3.18 | **<0.001** |
| Age (years) | 1.06 | 1.05, 1.08 | **<0.001** |
| Sex (ref: Male) |  |  |  |
| Male | — | — |  |
| Female | 1.06 | 0.83, 1.35 | 0.635 |
| Education level |  |  |  |
| Primary or below | — | — |  |
| Middle school | 1.32 | 1.03, 1.69 | **0.027** |
| High school | 1.19 | 0.93, 1.53 | 0.170 |
| College or above | 1.26 | 0.95, 1.66 | 0.104 |
| Marital status |  |  |  |
| Married | — | — |  |
| Widowed | 0.87 | 0.64, 1.19 | 0.381 |
| Divorced/Single | 0.92 | 0.67, 1.28 | 0.624 |
| BMI (kg/m²) | 1.03 | 0.99, 1.07 | 0.135 |
| Waist circumference (cm) | 1.00 | 0.98, 1.01 | 0.800 |
| Smoking history |  |  |  |
| No | — | — |  |
| Yes | 1.15 | 0.93, 1.43 | 0.191 |
| Alcohol consumption |  |  |  |
| No | — | — |  |
| Yes | 1.23 | 0.98, 1.54 | 0.076 |
| Physical activity |  |  |  |
| Low | — | — |  |
| Moderate | 0.99 | 0.82, 1.21 | 0.947 |
| High | 0.99 | 0.77, 1.27 | 0.918 |
| HTN duration (years) | 1.03 | 0.99, 1.06 | 0.108 |
| SBP (mmHg) | 1.01 | 1.01, 1.02 | **<0.001** |
| Triglycerides (mmol/L) | 1.04 | 0.94, 1.16 | 0.447 |
| HDL-C (mmol/L) | 0.69 | 0.45, 1.05 | 0.086 |
| LDL-C (mmol/L) | 1.05 | 0.96, 1.16 | 0.297 |
| Fasting glucose (mmol/L) | 1.00 | 0.91, 1.09 | 0.915 |
| Diabetes mellitus |  |  |  |
| No | — | — |  |
| Yes | 1.26 | 0.98, 1.62 | 0.072 |
| eGFR (mL/min/1.73m²) | 0.99 | 0.98, 1.00 | 0.137 |
| Dyslipidemia |  |  |  |
| No | — | — |  |
| Yes | 0.88 | 0.66, 1.17 | 0.380 |
| History of CHD |  |  |  |
| No | — | — |  |
| Yes | 1.51 | 0.91, 2.60 | 0.121 |
| Antihypertensive medication |  |  |  |
| No | — | — |  |
| Yes | 1.02 | 0.84, 1.25 | 0.816 |
| Urate-lowering therapy |  |  |  |
| No | — | — |  |
| Yes | 0.95 | 0.76, 1.19 | 0.649 |
| Aspirin use |  |  |  |
| No | — | — |  |
| Yes | 1.17 | 0.97, 1.41 | 0.106 |
| Statin use |  |  |  |
| No | — | — |  |
| Yes | 1.12 | 0.93, 1.34 | 0.230 |
| Abbreviations: CI = Confidence Interval, OR = Odds Ratio | | | |
| No. Obs. = 3,036; AIC = 3,153 | | | |
